# Glioblastomas are composed of genetically divergent clones with distinct tumourigenic potential and variable stem cell-associated phenotypes

**DOI:** 10.1007/s00401-013-1196-4

**Published:** 2013-10-24

**Authors:** Daniel Stieber, Anna Golebiewska, Lisa Evers, Elizabeth Lenkiewicz, Nicolaas H. C. Brons, Nathalie Nicot, Anaïs Oudin, Sébastien Bougnaud, Frank Hertel, Rolf Bjerkvig, Laurent Vallar, Michael T. Barrett, Simone P. Niclou

**Affiliations:** 1 NorLux Neuro-Oncology Laboratory, Department of Oncology, Centre de Recherche Public de la Santé (CRP-Santé), 84, Val Fleuri, 1526 Luxembourg, Luxembourg; 2 Clinical Translational Research Division, Translational Genomics Research Institute, Scottsdale, AZ USA; 3 Core Facility Flow Cytometry, Centre de Recherche Public de la Santé (CRP-Santé), Luxembourg, Luxembourg; 4 Genomics Research Unit, Centre de Recherche Public de la Santé (CRP-Santé), Luxembourg, Luxembourg; 5 Department of Neurosurgery, Centre Hospitalier de Luxembourg, Luxembourg, Luxembourg; 6 NorLux Neuro-Oncology, Department of Biomedicine, University of Bergen, Bergen, Norway

**Keywords:** Glioma, Ploidy, Clonal evolution, Cancer stem cell, Array CGH, Flow cytometry, Single cell array CGH

## Abstract

Glioblastoma (GBM) is known to be a heterogeneous disease; however, the genetic composition of the cells within a given tumour is only poorly explored. In the advent of personalised medicine the understanding of intra-tumoural heterogeneity at the cellular and the genetic level is mandatory to improve treatment and clinical outcome. By combining ploidy-based flow sorting with array-comparative genomic hybridization we show that primary GBMs present as either mono- or polygenomic tumours (64 versus 36 %, respectively). Monogenomic tumours were limited to a pseudodiploid tumour clone admixed with normal stromal cells, whereas polygenomic tumours contained multiple tumour clones, yet always including a pseudodiploid population. Interestingly, pseudodiploid and aneuploid fractions carried the same aberrations as defined by identical chromosomal breakpoints, suggesting that evolution towards aneuploidy is a late event in GBM development. Interestingly, while clonal heterogeneity could be recapitulated in spheroid-based xenografts, we find that genetically distinct clones displayed different tumourigenic potential. Moreover, we show that putative cancer stem cell markers including CD133, CD15, A2B5 and CD44 were present on genetically distinct tumour cell populations. These data reveal the clonal heterogeneity of GBMs at the level of DNA content, tumourigenic potential and stem cell marker expression, which is likely to impact glioma progression and treatment response. The combined knowledge of intra-tumour heterogeneity at the genetic, cellular and functional level is crucial to assess treatment responses and to design personalized treatment strategies for primary GBM.

**Electronic supplementary material:**

The online version of this article (doi:10.1007/s00401-013-1196-4) contains supplementary material, which is available to authorized users.

## Introduction

Tumour cell heterogeneity is a well-recognized hallmark of cancer and plays a crucial role in tumour growth, metastasis, angiogenesis and therapy resistance [18, 60]. Glioblastoma (GBM), the most common malignant brain tumour in adults, is characterized by extensive tumoural heterogeneity at both the cellular and the molecular levels [6]. It is also among the most lethal of all cancers with no efficacious treatment available. Historically, the moniker ‘multiforme’ relates to the exquisite diversity of histopathological features, which include a high degree of cellular and nuclear pleomorphism and the presence of numerous juxtaposed tumour regions, characterized by diffuse infiltration, pathological vasculature and necrosis that collectively define GBMs [32]. The clinical presentation and response to treatment can be variable between GBM patients, in part owing to a strong inter-tumoural heterogeneity as evidenced by the occurrence of different gene mutations affecting defined signalling networks [6, 11], the identification of several molecular subtypes [44, 55] including a Glioma CpG Island Methylator phenotype (G-CIMP) [40] and the variability in the methylation pattern of the *O*-6-methylguanine-DNA methyltransferase (MGMT) gene. The latter has been shown to modulate the treatment response to temozolomide [23]. Early reports revealed that different GBMs can present with different DNA contents [4, 47]; however, what has been less appreciated is the intra-tumoural molecular heterogeneity of these populations and their function as tumor-initiating cells.

Tumour cell heterogeneity within a given tumour may arise from clonal expansion and acquisition of mutations during tumour progression [21, 41] and/or under selective pressure (i.e. during therapy), favouring the survival of the best adapted clone with resistant properties. Such selection processes have been recognized in a number of cancers including leukaemia [16, 53], breast cancer [36, 46], pancreatic cancer [59] and renal carcinoma [17]. Accumulating evidence indicates that GBMs are also characterized by intra-tumoural heterogeneity at the genetic level. For instance, it is well established that in a subset of GBMs, the epidermal growth factor receptor mutant EGFRvIII is present only in a subpopulation of tumour cells [39], influencing surrounding cells by paracrine mechanisms [25]. The presence of area-specific clonal diversity in GBM patients was recently shown by FISH analysis [50] and by a combination of comparative genomic hybridisation (CGH) and expression profiling [52]. However, little is known about tumourigenicity and drug susceptibility of different GBM clones, nor about the extent of aneuploidization and whether aneuploidy is a cause or a consequence of cancer progression.

At another level the recent cancer stem cell (CSC) hypothesis postulates a hierarchical tumour organisation, where only a small subpopulation of tumour cells are thought to be responsible for sustaining tumourigenesis and hence cellular heterogeneity [56]. CSCs are defined by their increased tumourigenicity upon xenotransplantation, long-term self-renewal and differentiation capacity and have been proposed to be responsible for drug resistance and tumour relapse [33]. In GBMs, putative CSCs have been described as cells expressing membrane markers such as CD133, CD15, CD44, A2B5, integrin α6 or EphA2 receptor [5, 29, 42, 49, 51]. However, there is no consensus on the CSC phenotype and the identification and clinical significance of glioma CSCs remain controversial [2, 13, 19, 58]. Importantly, putative CSCs are not regularly interrogated for their genetic background and it has not been determined whether such cells can recapitulate the genetic heterogeneity seen in human GBMs.

In this study, we aimed to assess the ploidy profiles and copy number variations of GBMs at the inter- and intra-tumoural level using ploidy-based flow sort array comparative hybridization (FS-array CGH) [37, 45], including single cell array CGH. Combining high-precision flow sorting and high-definition genomics enabled us to establish molecular portraits of individual GBMs. We identify mono- and polygenomic GBMs and reveal the ploidy-based intra-tumoural genetic heterogeneity in a subset of GBMs. In combination with functional assays and phenotypic profiling, we show for the first time that (1) genetically divergent clones display different tumourigenic potential in vivo; (2) within polygenomic GBMs CSC associated marker expression is highly variable and distributed throughout genetically distinct clones. To our knowledge, this is the first in-depth analysis of ploidy-based intra-tumoural genetic heterogeneity in combination with CSC-associated phenotypes and tumourigenic potential in GBMs.

## Materials and methods

### Clinical glioblastoma samples

Thirty-six GBM samples were collected at the Neurosurgery Department of the Centre Hospitalier in Luxembourg (CHL), from patients that have given their informed consent. Collection and use of patient tumour material has been approved by the National Ethics Committee for Research (CNER) of Luxembourg. All biopsies were from grade IV GBMs (WHO grading system) based on neuropathological diagnosis. The student *t* test and Chi squared test were used to calculate association of the ploidy profiles with age and sex of the patients, respectively.

### Flow sort array comparative genomic hybridization (FS-array CGH)

Nuclei were isolated from fresh or liquid nitrogen flash-frozen patient biopsies and xenografts. Briefly, samples were minced in DAPI buffer [10 μg/ml DAPI in 146 mM NaCl, 10 mM Tris–HCl (pH 7.5), 0.2 % Nonidet P40] [43]. Nuclei were disaggregated subsequently with 20G and 25G needles and filtered through a 50- and a 30-μm mesh. Flow analysis and sort were carried out with an Influx cell sorter (BD Biosciences) or an Aria™ SORP flow cytometer (BD Biosciences) and the DAPI signal was excited with the UV laser. For xenograft analysis, tumour nuclei were recognized with the human-specific phycoerythrin-labelled anti-lamin A/C antibody (Santa Cruz, Biotech sc-7292 PE). DNA content was analysed with the MultiCycle (Phoenix Flow Systems) and ModFitLt (VSH) softwares.

For array CGH, DNA from sorted nuclei (at least 10,000 sorted nuclei) was extracted using the QIAamp Micro Kit (Qiagen) following the manufacturer’s protocol. For each hybridization, 100 ng of genomic DNA was amplified using the GenomiPhi amplification kit (GE Healthcare). Pooled female DNA from a commercial source (Promega) was used as a reference. Amplified samples and references (1 μg each) were digested with DNaseI and labelled with Cy-5 dUTP and Cy-3 dUTP, respectively, using the BioPrime labelling kit (Life Technologies). Prior to quantification, reactions were purified on a microcon YM30 to remove the excess of Cy-labelled dUTPs. All labelling reactions were assessed using a Nanodrop assay before mixing and hybridized to either 1,000,000, 400,000 or 244,000 feature human genome CGH arrays (Agilent Technologies) according to manufacturer’s instructions (CGH enzymatic protocol v6.2; Ref # G4410-90010).

Microarray slides were scanned using an Agilent 2565C DNA scanner, and the images were analysed with Agilent Feature Extraction version 10.5, using default settings.

Data were assessed with a series of quality control metrics and analysed using an aberration detection algorithm (ADM2) [31] implemented in the Genomic Workbench software package (Agilent). ADM2 identifies all aberrant intervals in a given sample with consistently high or low log ratios based on the statistical score derived from the average normalized log ratios of all probes in the genomic interval multiplied by the square root of the number of these probes. This score represents the deviation of the average of the normalized log ratios from its expected value of zero and is proportional to the height, *h* (absolute average log ratio), of the genomic interval and to the square root of the number of probes in the interval.

### Single nucleus array CGH

DNA of each sorted single nucleus was amplified by whole genome amplification (WGA) using a modified version of the protocol described by Navin [36]. Single nuclei were sorted directly to the WGA4 Genome Plex Kit lysis solution (Sigma Aldrich) 10 μl/well in a 96-well plate with 1 nucleus/well. Empty wells were used as negative controls. WGA was performed following the manufacturer’s recommendations (WGA4 Genome Plex Kit Sigma Aldrich). 1 ng of female reference DNA (Promega) was amplified using the same method and used as reference in array CGH. Samples were hybridized to 8 × 60,000 features Agilent human genome CGH array.

### Ploidy analysis combined with cell membrane phenotyping and viable cell sort

Patient biopsies and xenografts were minced with scalpels and dissociated with MACS Neural Tissue Dissociation Kit (P) (Miltenyi, 130-092-628) following the manufacturer’s instructions. Single cell suspensions were incubated with Hoechst 33342 (5 μg/ml, Bisbenzimide, Ho342; Sigma) at 37 °C in pre-warmed DMEM, containing 2 % FBS, 10 mM HEPES pH 7.4 and DNAse I (10 μg/ml; Sigma) at 1 × 10^6^ cells/ml for 120 min with gentle agitation on a shaker. No Hoechst efflux inhibitors were needed for ploidy assessment as GBM tumour cells do not possess efflux properties [19]. Since in the brain only endothelial cells efflux the Hoechst dye, CD45^+^/CD44^+^ haematopoietic cells were used as the internal diploid control. After washing, cells were resuspended in ice-cold HBSS 2 % FBS, 10 mM HEPES pH 7.4 buffer (100 μl/test). Prior to flow cytometry, cells were incubated with LIVE/DEAD^®^ Fixable Dead Cell Stains (Life Technologies; 1 μg/ml) and appropriate preconjugated antibodies for 30 min at 4 °C in the dark (antibodies are listed in supplementary Table 3). Data acquisition was performed on a FACS Aria™ SORP cytometer (BD Biosciences) and the Hoechst signal was excited with the UV laser. Data acquisition and analysis were done with DIVA software (BD Biosciences). Histograms were prepared with the FlowJo software. For sorting experiments, cells were collected in cold spheroid medium and cultured as described for tumour spheroids (spheroid medium in agar pre-coated plates). Sorted cells were plated at 10,000 cells/well in agar-coated 16-well plates to allow spheroid formation.

### Flow cytometer settings

The Influx cell sorter (BD Biosciences) was fitted with Solid-state lasers: 488 nm (200 mW); 355 nm (100 mW) and 640 nm (50 mW). The FACS Aria™ SORP cytometer (BD Biosciences), was fitted with a 632-nm (30 mW) red laser, a 355 (60 mW) UV laser, a 405-nm (50 mW) violet laser and a 488-nm (100 mW) blue laser. The DAPI and Hoechst dye were excited by the UV laser and fluorescence was collected in two channels: ‘UV-1’ 450/50 band-pass (BP) filter and ‘UV-2’ 660/40 long-pass (LP) filter. A LP 635 nm dichroic mirror was used to split the emission wavelengths. The flow cytometers were stabilized for at least 1 h before laser alignment and data acquisition. The Coefficient of Variation of the instrument (%CV) was routinely examined before each experiment. Routinely a 100-μm nozzle and window extension (WE) 5 were used for data acquisition and sorting. Cell acquisition and sorting were performed at 4 °C at a low fluidic sample speed. Data acquisition and analysis were done with DIVA software (BD Bioscience). To preserve the Hoechst profile and cell viability, all sorting experiments were performed directly after staining and under cold conditions.

### Orthotopic GBM xenografts based on organotypic biopsy derived spheroids

Organotypic GBM spheroids from patient samples were prepared as previously described [19, 28] and maintained in DMEM medium, 10 % FBS, 2 mM l-glutamine, 0.4 mM NEAA and 100 U/ml Pen-Strep (all from Lonza) in agar pre-coated flasks for 7–10 days. Tumour xenografts were generated in NOD/Scid immunodeficient mice expressing enhanced green fluorescent protein (eGFP) [38]. Mice were anaesthetized with a mixture of ketamine (10 mg/ml) and xylazine (1 mg/ml) and fixed in a stereotactic frame (Narishige Group, Tokyo, Japan). Tumour spheroids with a diameter of 200–300 μm (5–6 spheroids/mouse) were implanted into the right frontal cortex using a Hamilton syringe (Hamilton, Reno, NV, USA). Animals were killed at the appearance of neurological symptoms and weight loss. All procedures were approved by the national authorities responsible for animal experiments in Luxembourg. To facilitate tumour monitoring in vivo T16 and T101 patient-derived cells were transduced with a DsRed expressing lentiviral vector.

### Cell culture

The GBM stem-like cell lines NCH421k, NCH465, NCH601, NCH660 and NCH644, kindly provided by Dr. Christel Herold-Mende (Department of Neurosurgery, University of Heidelberg [10], were cultured as non-adherent spheres in DMEM-F12 medium (Lonza) containing 1× BIT100 (Provitro), 2 mM l-glutamine, 30 U/ml Pen-Step, 1 U/ml Heparin (Sigma), 20 ng/ml bFGF (Miltenyi, 130-093-841) and 20 ng/ml EGF (Provitro, 1325950500). The GBM stem-like cell lines TB101 and TB107, kindly provided by Dr. Håkan Hedman (Umeå University, Sweden) were cultured in DMEM-F12 medium (Lonza) containing 1× B27 and 1× N2 supplements (Provitro), 2 mM l-glutamine, 30 U/ml Pen-Step, 1 U/ml Heparin (Sigma), 20 ng/ml bFGF (Miltenyi, 130-093-841) and 20 ng/ml EGF (Provitro, 1325950500). U87, U251 and U373 cells were cultured as adherent monolayers in DMEM containing 10 % FBS, 2 mM l-glutamine and 100 U/ml Pen-Strep (all from Lonza).

## Results

### Primary GBMs are either monogenomic or polygenomic

**Fig. 1:**
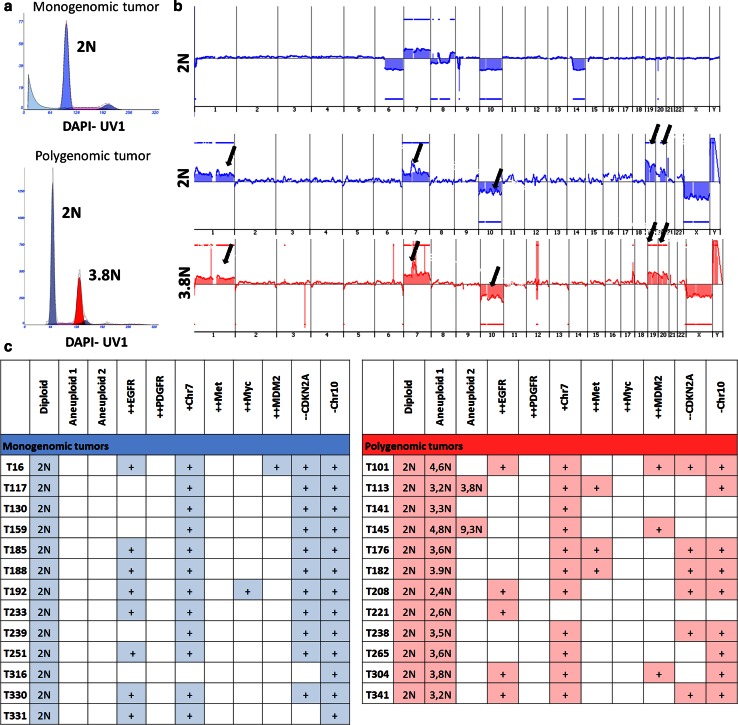
Mono- and polygenomic GBMs revealed by FS-array CGH analysis of GBM biopsies. **a** DAPI-based ploidy detection in isolated nuclei of GBM patient biopsies revealed inter-tumoural heterogeneity at the ploidy level. Examples of a monogenomic (T159, one G_1_/G_0_ DNA peak detectable and one G2/M peak) and a polygenomic tumour (T304, two G_1_/G_0_ DNA peaks, small G2/M peak) are shown. See supplementary Fig. 1a for flow cytometry gating strategies. Assuming that stromal cells were present in each patient biopsy the first peak recognized was considered as the diploid (2N) fraction (*blue*). Additional aneuploid fractions are shown in *red*. **b** Corresponding array CGH profiles of sorted nuclei from distinct DAPI peaks (shown in **a**) demonstrating the presence of tumour cells with typical GBM aberrations in all fractions (*blue* diploid, *red* aneuploid). Distinct populations from polygenomic tumours (T304) carried similar aberrations. *Arrows* indicate from *left* to *right*, +Chr1, ++EGFR, −Chr10, +Chr19, +Chr20. [++, amplification (log^2^ ratio >2); +, gain (log^2^ ratio >0.35); −, loss (log^2^ ratio < −0.35); −, deletion (log^2^ ratio < −3)]. See supplementary Fig. 2 for additional examples. **c** Summary of GBM biopsies analysed by FS-array CGH and major chromosomal aberrations identified. Monogenomic tumours in ‘*blue*’, polygenomic tumours in ‘*red*’. Of 36 GBMs analysed 23 (64 %) were monogenomic and 13 (36 %) were polygenomic. See supplementary Table 1 for additional samples

The development of genomic instability leading to the evolution of aneuploid cell lineages is a hallmark of many cancers and was early recognized to be associated with a poor prognosis [7, 26, 27, 34]. Therefore, difference in DNA content is a first layer of genomic individuality of clones where differences in ploidy can be used as a proxy for genetic heterogeneity. To assess the intra-tumoural clonal architecture and the extent of genetic heterogeneity in primary GBMs, we performed flow cytometric analysis based on DNA staining (i.e. using DAPI or Hoechst) on 36 patient biopsies to discriminate the DNA content present in individual nuclei of a complex tumour sample (supplementary Fig. 1). Assuming that each patient biopsy also contained normal stromal cells, the first histogram peak in the DAPI channel typically was considered diploid and each successive peak represented either the diploid 4N (G_2_/M)fraction (Fig. 1a, top) or a distinct aneuploid population within the tumour (Fig. 1a, bottom; supplementary Fig. 1a). In this context, we considered ploidies to be unique when differences in the DNA index (DI) of DAPI stained nuclei were ±0.1 (differences in ploidy of ±0.2N). Analysis of 36 tumour biopsies showed that GBMs display heterogeneous profiles based on DNA content and corresponding copy number variation as determined by FS-array CGH (Fig. 1a, b; supplementary Table 1). 23 GBMs (64 %) were monogenomic and 13 GBMs (36 %) were heterogenomic (polygenomic) having at least one additional aneuploid peak (Fig. 1c; supplementary Table 1).In two patients (T113 and T145) a second aneuploid peak was detected (Fig. 1c; supplementary Table 1). We found no association between the ploidy profile and the patient age (student *t* test, *p* = 0.39) and sex (Chi squared test, *p* = 0.63) (supplementary Table 1). Of note, five patients underwent a second surgery after recurrence, of which four tumours were monogenomic and one was polygenomic in the primary biopsy, which upon recurrence retained their ploidy level (supplementary Table 1).

To assess the genomic profiles of the ploidy-based clones in patient biopsies, each individual DNA peak was FACS-sorted and sorted nuclei were analysed with array CGH to obtain high-definition clonal profiles of the copy-number aberrations in each tumour genome (Fig. 1b; supplementary Fig. 2). The aberrant genomic intervals in each population were determined by the aberration detection algorithm ADM2 [31]. All the monogenomic tumours exhibited genetic aberrations typical of a GBM genome (Fig. 1b upper panel, additional examples in supplementary Fig. 2a), confirming the presence of tumour cells in each patient biopsy. In polygenomic tumours, all sorted aneuploid clones purified from patient biopsies also exhibited a typical GBM profile [11] (Fig. 1b lower panel; supplementary Fig. 2b ‘red’). Interestingly, in all cases of polygenomic samples the FACS-sorted diploid peak also exhibited genomic aberrations (Fig. 1b middle panel; supplementary Fig. 2b ‘blue’). The lower resolution of aberrations detected by array CGH in certain cases in the diploid fractions compared with aneuploid fractions were likely due to the presence of admixed non-tumour cells within the same DAPI peak, thereby diluting the tumour clone and obscuring tumour-specific genomic aberrations (e.g. T101GBM; supplementary Fig. 2b).

For all monogenomic and polygenomic tumours analysed, aberrations were seen at the level of large-scale structural aberrations such as gains or losses of whole chromosomes or chromosome arms as well as at the level of more focal events including high-level (log^2^ ratio >1) amplifications and homozygous(log^2^ ratio < −3) deletions. Importantly, the number of ADM2-defined intra-chromosomal copy-number aberrations in sorted populations varied widely between individual patient biopsies (from 10 in several samples up to 129 in the aneuploid fraction of T221) (supplementary Fig. 2) illustrating the individuality of GBM genomes. Although the same GBM associated genes were often targeted by recurrent aberrations (e.g. amplification of EGFR, homozygous deletion of CDKN2A or chromosome 10; Fig 1c), we did not detect any common breakpoints between different tumours for a given targeted gene, highlighting the uniqueness of each patient’s tumour genome (not shown). Moreover, we did not detect any obvious correlation between typical GBM genetic aberrations and the ploidy profiles of the tumours (Fig. 1c).

In summary, we show that individual GBM patients harbour unique tumour genomes both at the level of their DNA content and at the level of their chromosomal structure. A small majority of GBMs (64 %) were found to be diploid by flow cytometry, versus 36 % of polygenomic tumours containing diploid and aneuploid tumour cell populations. Importantly, recurrence of the disease did not necessarily involve aneuploidization and appearance of new divergent clones.

### Monogenomic tumours consist of aberrant pseudodiploid cells admixed with normal stromal cells

**Fig. 2:**
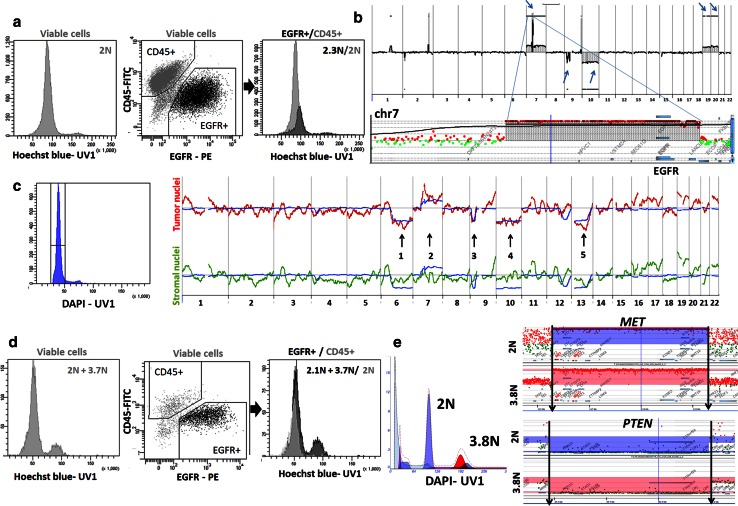
Monogenomic tumours always contain an aberrant pseudodiploid population admixed with normal stromal cells, whereas polygenomic tumours additionally contain one or more aneuploid fractions. **a** Hoechst-based profiling on viable cells from a monogenomic GBM (T251) showing a wide DNA peak (*left*). Tumour cells were recognized as EGFR^+^ (‘*black*’) whereas hematopoietic stromal cells were identified as CD45^+^ (‘*grey*’) (*middle panel*). Ploidy analysis of EGFR^+^ tumour cells showed a pseudodiploid peak with a significant shift in the DNA content compared with the CD45^+^ diploid control (2.3N versus 2N peak in *right panel*). See more examples in supplementary Fig. 4a. **b** Array CGH profile of T251 confirms typical GBM rearrangements, contributing to the pseudodiploid DNA profile. *Arrows* indicate (from *left* to *right*) EGFR amplification on trisomy 7, deletion of CDKN2A/B, monosomy 10, trisomy 19 and 20. *Inset* shows a detailed view of the EGFR amplicon on chromosome 7. **c** Array CGH on single nuclei. Single nuclei of a monogenomic GBM (T16) were individually collected from the same DAPI peak (*left panel*), amplified and probed by array CGH (*right panel*). 14/16 sorted nuclei were detected as tumour cells (*red*), 2/16 were stromal cells with no aberrations detected (*green*). *Blue line* corresponds to ‘bulk’ tumour nuclei (non amplified DNA from millions of unsorted nuclei). *Numbers* indicate large chromosomal losses and gains detected only in ‘bulk’ and isolated tumour nuclei. **d** Hoechst-based profiling on viable cells from a polygenomic GBM (T238) which contained two ploidy peaks (2N and 3.7N, *left panel*). Ploidy analysis of EGFR^+^ tumour cells (*middle panel*; ‘*black*’) versus haematopoietic CD45^+^ stromal cells (‘*grey*’) confirmed the presence of two distinct tumour clones (2.1N and 3.7N, *right panel*). **e** In-depth comparison of two tumour clones within a polygenomic GBM (T176) showing identical chromosomal breakpoints in the two fractions. The MET amplicon and the PTEN homozygous deletion are shown for the 2N (*blue*) and 3.8N fraction (*red*). *Arrows* indicate the borders of the deletion/amplicon, respectively. See supplementary Fig. 3 for more examples. See supplementary Fig. 1 for flow cytometry gating strategies

Since all of the monogenomic tumours displayed only one diploid DNA peak, we assumed that they were composed of an abnormal tumour cell population with the approximate (eu)ploidy of normal stromal cells. Indeed all monogenomic tumours were confirmed by array CGH to contain a rearranged genome (Fig. 1a; supplementary Fig. 2a). To distinguish admixed tumour and stromal cell populations within one DNA peak, we performed ploidy analysis based on Hoechst staining combined with cell membrane phenotyping in viable cells (Fig. 2a). Hoechst 33342 is regularly used for assessment of efflux properties [Side Population (SP) phenotype] in (cancer) stem cells and we have previously shown that the gating strategy for the SP phenotype relies strongly on the ploidy of tumour cells [20]. Therefore, to assess ploidy in viable cells carrying the SP phenotype it is essential to inhibit Hoechst efflux. This does not, however, apply to GBM, since we [19] and others [9] have recently shown that GBM tumour cells do not carry efflux capacities. Indeed in GBM tissue only stromal endothelial cells were found to display the SP phenotype [19]. Therefore, Hoechst staining could be used directly for ploidy assessment and FACS-sorting of viable cells. Interestingly, in all tested biopsies EGFR^+^ tumour cells displayed a significantly higher DI than stromal haematopoietic cells (CD45^+^/CD44^+^; Fig. 2a; supplementary Fig. 4a). Tumour cells could thus be designated as “pseudodiploid” [36] as they carry an apparently diploid genome (by DNA content) despite harbouring multiple chromosomal aberrations including amplifications and deletions (see T251 GBM example on Fig. 2b arrows and inset).

To further confirm an admixture of tumour and stromal cells within monogenomic biopsies, we performed array CGH on single nuclei sorted from the same uniform DAPI peak of the T16 patient biopsy (Fig. 2c). DNA of each sorted single nucleus was amplified by WGA before array CGH was performed. Ultra-diluted and amplified female DNA was used as a reference. For the T16 GBM, 16 independent nuclei from the detected DNA peak were analysed and compared with the “bulk” sorted DNA peak. A majority of the single nuclei (14/16) showed aberrations also detected in the bulk tumour peak (Fig. 2c, red versus blue line), but occasional nuclei (2/16) displayed a flat profile without any of the aberrations detected in the bulk tumour sample (Fig. 2c, green), representing admixed stromal cells in the biopsy material. Although the resolution of single nucleus array CGH is limited owing to the very small amount of starting DNA, our data indicate that the technique allows to successfully assess clonal diversity within one DNA peak (Fig. 2c, arrows indicating the ‘major’ chromosomal aberrations established previously in the ‘bulk’ tumour).

Thus by applying two different methods we demonstrate that monogenomic diploid GBMs consist of an aberrant pseudodiploid tumour cell population admixed with normal stromal cells. Hoechst-based analysis combined with phenotyping in viable cells and single nucleus sort combined with array CGH analysis are powerful tools to analyse the heterogeneity of cells present within a complex tumour sample displaying similar ploidy level.

### Genomic profiling of GBM patient samples reveals a clonal evolution process towards aneuploidy

Since all polygenomic GBMs contained a diploid fraction, we first confirmed the presence of tumour cells in the diploid fraction and validated the purity of the nuclei sort by applying Hoechst-based ploidy analysis (see example of T238 GBM on Fig. 2d). EGFR^+^ tumour cells showed two distinct clones with varying ploidy (Fig. 2d, right). Similar to the monogenomic GBMs, tumour cells within the diploid peak appeared pseudodiploid, with a shift at the DNA level compared with diploid control cells, further confirming the genomic aberrations present in this fraction.

To understand the clonal progression between diploid and aneuploid clones in GBM we performed an in-depth comparison of aberrations present in diploid and aneuploid tumour cells. We have shown in Fig. 1b and supplementary Fig. 2b that aberrations detected by array CGH in the diploid fractions were also present in the aneuploid clones of the same tumours, suggesting that tumour populations are clonally related. The same chromosomal aberrations were detected at the level of large-scale structural aberrations (e.g. chromosomal loss) as well as at the level of more focal events (high level amplifications and homozygous deletions) (Fig. 1b, additional examples on supplementary Fig. 2b). Moreover, different clones present within one patient biopsy carried the exact same chromosomal breakpoints for any given aberration. Figure 2e shows the chromosomal breakpoints for the PTEN deletion and the MET amplicon in the T176 GBM (additional examples on supplementary Fig. 3). This was also true for all other polygenomic GBMs analysed interrogating, e.g. amplifications of MDM2, CDK4, EGFR or deletions of CDKN2A and PTEN (supplementary Fig. 3).

Taken together, our data indicate that aneuploid GBM cells carry the genomic aberrations already present in the pseudodiploid tumour cells. Since the genetic aberrations were shared between clones of the same biopsies, and at the same time aberrations varied widely between patients, it can be inferred that tumour populations within one patient biopsy are clonally related and likely share a common ancestor. By applying the principle of non-reversibility of acquired somatic events and by taking into account that all GBMs contained a pseudodiploid fraction, our data suggest that aneuploidization is a late event in GBM, which occurs once initial driver aberrations/mutations have been acquired by the tumour genome.

### Genomic heterogeneity of GBMs is retained in organotypic biopsy spheroid-based xenografts

**Fig. 3:**
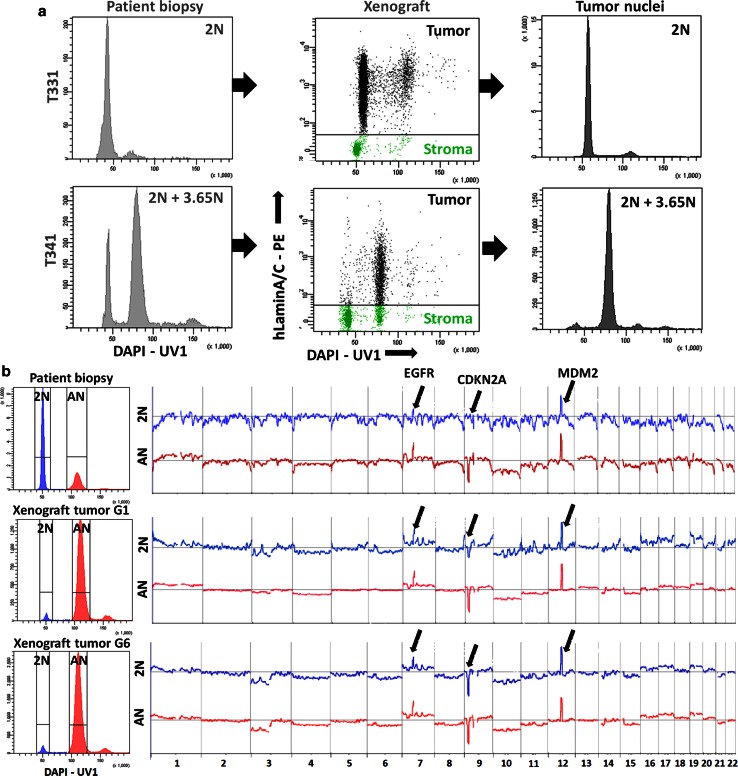
Genetic heterogeneity is retained in spheroid-based xenografts. **a** Monogenomic (T331) and polygenomic (T341) GBMs were used for derivation of spheroid-based xenografts in NOD/Scid mice. In xenografts, tumour nuclei (‘*black*’) were recognized by human-specific laminA/C positivity (*middle panel*). DAPI staining of human nuclei in xenografts showed that tumours retained the ploidy detected in the parental biopsy (*right*). See supplementary Fig. 4c, d and supplementary Table 2 for more examples. **b** The polygenomic T101 GBM was serially transplanted in NOD/Scid mice (*G1* generation 1, *G6* generation 6). Diploid (2N, *blue*) and aneuploid (AN, *red*) tumour clones detected by nuclear DAPI staining were sorted from the parental biopsy and corresponding xenografts for array CGH analysis. Clones retained their genetic profile upon serial transplantation (see highlighted aberrations). *Arrows* indicate (from *left* to *right*), amplification of EGFR, deletion of CDKN2A/B and amplification of MDM2

To characterize the phenotypic properties of divergent genetic clones present within the same GBM we established intracranial xenografts in NOD/Scid mice from a number of mono and polygenomic GBMs (supplementary Table 2). We and others have previously shown that xenografts based on organotypic spheroids closely maintain the genetic, phenotypic and behavioural profiles of the parental patient tumours [15, 28, 57]. DAPI-based analysis revealed that the heterogeneity present at the ploidy level in patient biopsies was recapitulated in the respective xenografts (Fig. 3a; supplementary Table 2). Importantly, we were able to discriminate tumour nuclei (‘black’) from the stromal compartment (‘green’) by human-specific lamin A/C positivity (Fig. 3a). Stromal nuclei were used as an internal diploid control as human and mouse nuclei have the same DNA index (supplementary Fig. 4b). An example of a monogenomic (T331) and a polygenomic (T341) tumour is depicted in Fig. 3a. The presence of distinct pseudodiploid and aneuploid clones was further confirmed with Hoechst-based ploidy discrimination in eGFP expressing NOD/Scid mice in multiple GBM xenografts (supplementary Fig. 4c, d). The exact recapitulation of the ploidy clones from patient biopsies in spheroid-based xenografts was true for all tumours analysed, except for T16 GBM, where the aneuploid population was only detected in the xenografts (supplementary Table 2). This may be explained by a very small aneuploid population in the biopsy or, more likely, by the spatial heterogeneity of the tumour, where the aneuploid fraction was present only in the specimen part used for spheroid derivation and not in the part used for initial ploidy analysis of the parental tumour. However, the possibility of cell aneuploidization occurring in the xenograft cannot be completely ruled out.

We then determined whether in xenografts divergent clones from polygenomic tumours retained the genetic aberrations present in the patient biopsies by applying DAPI-based FS-array CGH. Both diploid (2N in blue) and aneuploid (AN in red) clones were purified from the patient biopsy (T101) and from two serially transplanted xenografts (generation 1 and 6) (Fig. 3b). Diploid and aneuploid clones remained remarkably similar upon serial xenotransplantation and retained their initial ploidy as well as all major aberrations and identical chromosomal breakpoints as seen in the parental biopsy (Fig. 3b, arrows).

Taken together, we show that GBM ploidy-based genomic heterogeneity can be recapitulated in organotypic spheroid-derived xenografts. Importantly, tumour cells retain their initial ploidy and the genetic aberrations upon serial transplantation in the mouse brain. This is in contrast to in vitro cultures of patient-derived glioma cell lines, which are known to undergo strong selection, aneuploidization and acquisition of new genetic aberrations in culture [30]. Of note in this context, GBM stem-like cell lines grown under serum-free conditions were found, similar to classic adherent glioma cultures, to be exclusively aneuploid, with only one out of seven lines (TB107) containing both diploid and aneuploid cells (supplementary Table 2).

### Pseudodiploid and aneuploid cells are capable of initiating tumours in mice but show differential growth characteristics

**Fig. 4:**
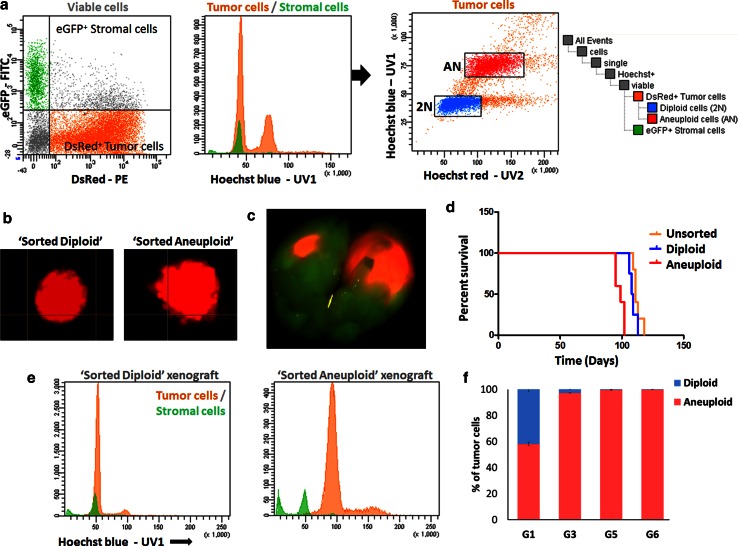
Distinct ploidy-based tumour clones show differential growth characteristics in vivo. **a** DsRed^+^ polygenomic GBM (T16) established in eGFP^+^ mice was used for Hoechst-based ploidy detection in viable cells and for sorting of distinct tumour clones. See supplementary Fig. 1b for the gating strategy. Tumour cells were recognized as DsRed^+^ eGFP^−^ cells (‘*orange*’) and gated out from DsRed^−^ eGFP^+^ mouse stromal cells (*green*) (*left panel*). Two distinct clones were detected within the tumour population (*red peaks in middle panel*) in addition to the diploid stromal cells (*green*). The diploid (2N, *blue*) and aneuploid (AN, *red*) tumour cells were sorted (*right panel*) and used for subsequent analysis. **b** Both diploid and aneuploid tumour clones formed spheroids in vitro within 10 days of culture. **c** Representative image of intracranial tumour developed from DsRed^+^ spheroids in eGFP^+^ mice. **d** Mice implanted with the aneuploid clone (*red*) died significantly earlier compared with those carrying the diploid clone (*blue*) or the bulk tumour (*orange*) (*p* = 0.006). No significant difference was detected between sorted diploid and unsorted bulk tumour (*p* = 0.173) (Kaplan–Meier plot, *n* = 5 per group). **e** Hoechst-based ploidy analysis of xenografts derived from sorted clones indicating that the implanted clones retained their initial pre-sort ploidy. **f** Serial transplantation of T16 spheroids of bulk tumour showing an overgrowth of aneuploid cells in late generations. The ratio between diploid and aneuploid cells in the tumour cell compartment is indicated for successive generations (**g**)

We then assessed whether aberrant clones were independently capable of initiating tumours in mice. Using Hoechst-based discrimination of viable cells, distinct tumour clones (pseudodiploid and aneuploid) were isolated from a polygenomic GBM transduced with DsRed expressing lentiviral vectors (Fig. 4a, T16 xenograft). Diploid (2N, blue) and aneuploid (AN, red) tumour clones were separately tested for their spheroid formation capacity in vitro and tumourigenic potential in vivo. Both diploid and aneuploid cells formed spheroids in vitro with a similar efficacy and similar size within 10 days of culture (Fig. 4b). To assess the tumourigenic potential in vivo, six spheroids of each clone were implanted intracranially into eGFP^+^ NOD/Scid mice (*n* = 5 per group). All mice developed tumours as observed by fluorescence imaging (Fig. 4c). However, mice carrying aneuploid spheroids displayed significantly shorter survival times compared to those engrafted with either diploid or unsorted bulk spheroids (98.6 ± 1.56 versus 109 ± 1.47 and 112.4 ± 1.53 days, respectively, *p* = 0.021 2N versus AN, *p* = 0.0002 AN versus bulk unsorted) (Fig. 4d). Subsequent Hoechst-based ploidy analysis of the tumours showed that within one xenograft generation clones were genetically stable and had retained their initial ploidy (Fig. 4e). Interestingly, during serial transplantation of unsorted T16 spheroids the aneuploid cells eventually outgrew the diploid population (Fig. 4f), confirming the differential in vivo growth potential of the two distinct populations. A similar behaviour was seen for T238 with a decrease in the diploid population and a shortened survival time over three serial transplantations (not shown).

In summary, our data suggest that both pseudodiploid and aneuploid tumour clones can establish tumours in mice; however, the aneuploid clones lead to more aggressive tumours. The aneuploid fraction appears to have a growth advantage over the pseudodiploid population, which is also reflected by the clonal outgrowth and apparent selection over time. The present in vivo GBM model based on serial transplantation of biopsy spheroids could thus be considered as a proxy for the dynamic behaviour of distinct individual clones in a patient tumour.

### Cancer stem cell-associated marker expression does not define a genetically uniform clone

**Fig. 5:**
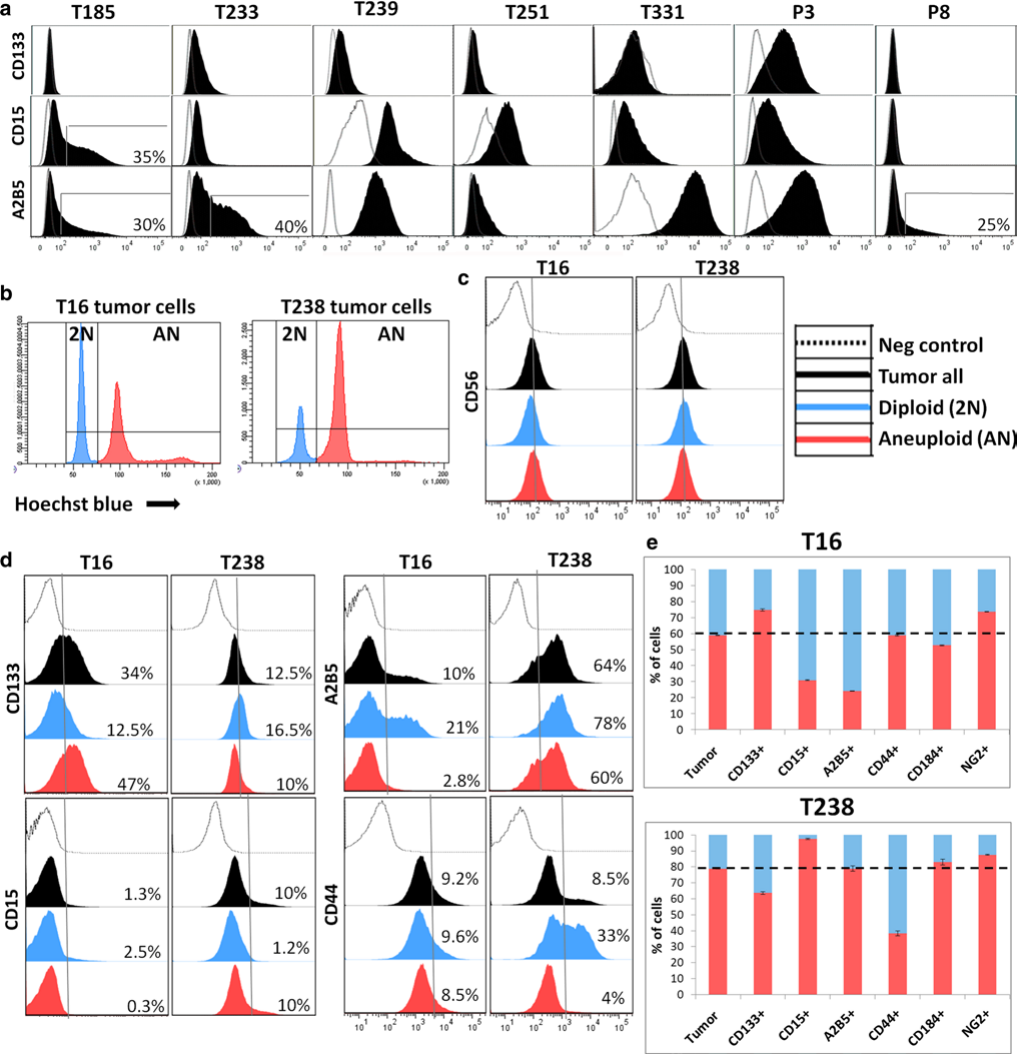
Genetically divergent clones display variable cancer stem cell-associated markers. **a** CSC-associated marker expression (CD133, CD15 and A2B5) as determined by flow cytometry in monogenomic GBM xenografts, showing a high variability between different GBMs. Expression (*black*) is displayed compared with negative controls (*grey line*). Intra-tumoural heterogeneity in some GBMs is presented as percentage of cells expressing high levels of CSC-associated markers. **b** Two polygenomic tumours (T16, T238) chosen for Hoechst-based ploidy profiling combined with cell membrane marker expression. **c** CD56 expression is similar in diploid and aneuploid clones of T16 and T238 GBMs. **d** Differential expression of CD133, CD15, A2B5 and CD44 in diploid and aneuploidy clones of T16 and T238 GBMs. Intra-tumoural heterogeneity is detected for all markers in the bulk tumour cells (‘*black*’). The discrimination between diploid (‘*blue*’) and aneuploid (‘*red*’) cells showed that marker expression varied between the two clones. The expression profiles and the percentage of positive/strongly positive cells for a given marker are displayed for each clone compared with negative controls (*grey line*). **e** The ratio of diploid versus aneuploid cells within a given marker-positive population is indicated, in comparison with the ratio detected in the bulk tumour cells (*dotted line*), indicating a high variability in marker expression between the genetically distinct clones. See supplementary Fig. 1b for the gating strategy

In GBMs several markers have been proposed to enrich for putative glioma CSCs including CD133, CD15, CD44 and A2B5 [42, 49, 51]. However, CSC discrimination approaches largely rely on cell membrane phenotyping without taking into account the genetic characteristics of the tumour cell subpopulation. Based on the differential tumourigenicity of pseudodiploid and aneuploid GBM clones, we asked whether the stem-like associated marker expression was specific to the aneuploid cell population. We, therefore, combined cell membrane phenotyping with Hoechst-based ploidy measurement in our GBM xenografts. As most of the putative glioma CSC associated markers (e.g. CD133, CD15, A2B5 and CD44) are not unique to tumour cells but are also expressed by stromal cells [19], we used spheroid-based xenografts in eGFP^+^ NOD/Scid mice for a distinct analysis of pure tumour cell populations. In agreement with earlier studies, different GBMs displayed a strong heterogeneity in the number of marker-positive cells, e.g. CD15 or A2B5 positive cells ranged from 0 to 100 % in different GBM samples (Fig. 5a). Additionally, some biopsies displayed a heterogeneous staining intensity with the presence of low and high expressing cells. This was equally true for monogenomic (e.g. CD15 expression in T185 GBM, Fig. 5a) and polygenomic tumours (Fig. 5d, bulk tumour represented by ‘black’ histogram). To gauge the influence of genetic heterogeneity on CSC-associated marker expression profiles, we closely analysed two polygenomic GBMs (T16 and T238) containing distinct genetic clones with significant amounts of diploid (2N, blue) and aneuploid (AN, red) tumour cells (Fig. 5b). Several cell membrane markers such as CD56 (Fig. 5c), CD90 and CD29 (not shown) were found to be expressed at similar levels in diploid and aneuploid clones. Interestingly, a subset of cell membrane markers was differentially expressed between clones in one or both cases contributing to a heterogeneous intra-tumoural expression profile in the tumour bulk (Fig. 5d, ‘black’). For example in the T16 pseudodiploid fraction, A2B5 was strongly enriched in the diploid fraction (21 %) compared with the aneuploid population (2.8 %), whereas CD133 was enriched in the aneuploid fraction (47 versus 12.5 % in the diploid fraction) (Fig. 5d). However, the inverse was true for CD133 expression in the T238 tumour (16.5 % positive cells in diploid versus 10 % in aneuploid fraction), indicating that the expression of a particular marker could not be directly correlated with cellular ploidy. Similarly, CD15^+^ cells were present almost exclusively in T16 diploid (2.5 versus 0.3 %) and T238 aneuploid clones (10 versus 1.2 %) (Fig. 5d). Other markers such as CD44 (Fig. 5d) and CD184 (not shown) varied in distinct clones only in one of the patient biopsies. While the two distinct clones were present in all marker-expressing populations analysed, the proportion of each of the clones compared with the respective tumour bulk significantly changed for each marker (Fig. 5e). This indicates that the isolation of tumour cells based on CSC associated marker expression leads to genetically heterogeneous subpopulations with different tumourigenic potential.

Taken together, we show that distinct ploidy-based tumour clones in polygenomic GBMs are heterogeneous at the phenotypic level and that CSC identification based on marker expression may be biased due to the changing ratio of genetically distinct clones. We, therefore, suggest that functional assays on tumour cell subpopulations separated by marker expression should consider the genetic landscape of the cells.

## Discussion

Tumour progression and resistance to therapy have been associated with tumour heterogeneity both at the genetic and the phenotypic level. Inter- and intra-tumoural heterogeneity have major implications for therapy and pose serious challenges for the rational design of effective treatment principles. Specifically, personalized medical approaches will be hampered if the heterogeneity of the individual tumour is not considered. The existence of topographic genetic heterogeneity has been elegantly shown in cancers of pancreas and breast using various multi-sampling approaches [36, 59]. The presence of region-specific aberrations and gene expression profiles has also recently been documented within GBM patient biopsies [50, 52, 54]. Here we report on an additional level of inter- and intra-tumoural heterogeneity in GBM biopsies based on ploidy analysis, genome wide copy number variation and phenotypic marker expression. We also provide evidence that ploidy-based clones display differential aggressiveness and variable CSC associated marker profiles.

Genomic instability and the evolution of aneuploid cell lineages are a hallmark of many cancers; therefore, DNA content is an important measure of intra-tumoural genetic heterogeneity, which is, however, often neglected because of not being directly visible in the analysis of complex tumour samples. Distinct genetic clones identified based on their DNA content revealed that a significant number (64 %) of GBMs are monogenomic and propagate by pseudodiploid tumour cells, even in recurrent tumours. Interestingly, as for other tumour types [17, 59] many GBM biopsies (36 %) contained several aberrant tumour clones at the ploidy level, revealing a high clonal complexity in GBM. Since in most cases we only had access to one biopsy from each patient tumour, the number of distinct clones represents the minimal number of cancer genomes per GBM. It is possible that additional aneuploid populations may be identified from distinct biopsy sites using a topographic sampling approach.

To our knowledge, this is the first in depth study addressing GBM intra-tumoural genetic heterogeneity by DNA content and down to the single-cell level. Genomic analysis of cellular subpopulations that differ in ploidy has been performed in other cancers, including breast [37], cervical, colorectal [14], prostate and pancreas [3, 45]. Compared with other genomic techniques, such as gene expression arrays and next-generation sequencing, which measure signals from a complex mixture of cell types, our approach has the advantage to dissect this complex mixture into distinct tumour clones and stromal cells. Indeed, a major problem that confounds the comprehensive characterization of tumour progression mechanisms is the presence of mixed cell populations. FACS sorting of distinct tumour clones combined with array CGH, allows high-resolution analysis of genomic aberrations present in individual fractions within each patient sample. By using purified populations we could objectively identify tumour-specific copy-number aberrations, including homozygous deletions and high-level amplifications, regardless of tumour cell content in each biopsy for the aneuploid clones. It is important to note that the difference in ploidy between diploid and pseudodiploid cells is below the resolution of standard methods and admixed cell populations with a uniform DNA content cannot be separated based on ploidy differences only. Therefore, we applied two different approaches to reveal the heterogeneity within one population determined at the ploidy level: (1) by combining FACS sorting and array CGH analysis of single nuclei we were able to confirm the heterogeneity present in diploid populations. (2) Using Hoechst-based ploidy discrimination combined with cell membrane phenotyping on living cells we confirmed the pseudodiploid nature of tumour cells, consistent with their genetic aberrations. Thus, in contrast to breast [37] and pancreatic cancer [3] the diploid populations in GBMs were aberrant and always contained tumour cells both in mono and polygenomic biopsies.

Aneuploidy in tumours was first observed over a century ago and has been recognized as a characteristic feature of cancer genomes [8, 22]. Most, if not all, cancer types show aneuploidization to a certain extent with the proportions varying between tumour types [12, 35]. It is still debated whether aneuploidy is a cause or a consequence of acquired genetic instability and the accumulation of somatic variants. Aneuploidy can be considered a mere benign side effect of cellular transformation, or alternatively a core element contributing to the growth and adaptability of cancer [24]. As monogenomic GBMs contained only a pseudodiploid tumour population and polygenomic GBMs were always composed of aberrant pseudodiploid and additional aneuploid cells, the aneuploidization appears to be a late event in GBM development. This is further supported by our observations showing that the majority of chromosomal breakpoints are shared between distinct clones within a particular biopsy. It appears that once the “classical” GBM aberrations are established in pseudodiploid cells (including, e.g. CDKN2A and PTEN loss, double minute amplifications of EGFR) they are retained in the aneuploid population. A similar conclusion was recently drawn from the computational analysis of genotyping and sequencing data from the GBMs analysed in the TCGA project [12]. Interestingly, the oncogenic fusion protein (FGFR-TACC fusion) recently detected in GBM has been shown to interfere with cell division and promote aneuploidy, suggesting that in this particular case, events that provoke aneuploidization may be a cause of cancer [48]. From our data it appears that GBM recurrence does not necessarily involve aneuploidization and appearance of new divergent clones. This is most probably due to the fact that in GBMs a high number of invasive tumour cells are retained in the brain after surgery, which leads to fast recurrence without the necessity of strong clonal selection.

Previous studies addressing intra-tumour heterogeneity did not investigate the tumourigenic potential of different clones. Here we show that the clonal diversity of GBM biopsies was transplantable in spheroid-based xenografts, suggesting that both aberrant diploid and aneuploid populations participated in spheroid formation and subsequently engrafted in mice upon orthotopic implantation, while maintaining their genetic aberrations. This finding validates the clinical relevance of the patient-derived spheroid xenograft model system where the full clonal and genomic complexity of the parental GBM is maintained. This is in striking contrast to other models including adherent cell lines and those grown as neurospheres in serum-free medium, which almost all had lost the pseudodiploid clones. Interestingly, in our xenografts each GBM clone was also able to independently form a tumour in vivo; however, the aneuploid-derived tumours developed faster. This was consistent with the observation from our serial transplantation studies, where aneuploid cells overtook the pseudodiploid population over time and may at least partially explain the decreased survival time of mice observed upon serial transplantation. Thus, in analogy to other tumour types the aneuploid fraction appears to be the more aggressive clone, a phenomenon that was early recognized in many tumors [1, 26, 34]. Our in vivo transplantation model, which allows a temporal follow-up of the tumours, may, therefore, be considered a proxy for the clinical behaviour of the tumours, particularly if more than one clone is present. In this context it would be interesting to determine if GBM aneuploidy can be linked to poor patient outcome, as has been shown for other cancers.

Currently, a lot of work is focusing on targeting putative CSCs in GBM, although the identification and significance of glioma CSCs remains controversial [2, 13, 58] and no consensus has been reached regarding appropriate glioma CSC markers. Furthermore, there is a considerable debate on whether CSCs represent a distinct tumour subpopulation or merely reflect a particular phenotypic state that most tumour cells can adopt [56]. In this context, tumour heterogeneity is often analysed at the phenotypic level without taking into account the genetic background of the tumour cells. For instance, it has been postulated that upon transplantation putative CSCs are able to recapitulate the phenotypic heterogeneity of the tumours from which they derive, yet it remains to be determined whether CSCs are a genetically uniform and identifiable subpopulation of tumour cells and whether CSC transplantation also recapitulates the genetic heterogeneity of the parental tumour. We have recently shown the importance of adequately discriminating stromal and tumour populations in the search for putative glioma CSCs [19, 20], e.g. the SP phenotype, a marker of CSCs in some cancers—although detected in human GBM, was found to be absent from GBM tumour cells upon separation of tumour and stromal compartments [19]. Only endothelial cells displayed the SP phenotype and this was independent of the genetic makeup, ploidy and marker expression of the tumour, thus indicating that SP is not a marker for putative glioma stem cells. In the present study, we used ploidy-based genetic analysis combined with tumour/stroma discrimination and CSC-associated marker profiling and found that glioma CSC marker expression does not define a genetically unique tumour clone. Our data suggest that the clonal evolution and CSC models of tumour progression both contribute to the rationalisation of tumour heterogeneity at different levels. Importantly, as the ratio of genetic clones varies in putative CSC populations depending on the marker used, the isolation of CSCs based only on phenotypic profiling changes the balance between genetically divergent clones with distinct functional properties (e.g. tumourigenic potential). Therefore, we propose that CSC-associated populations should be interrogated at the genetic level including the ploidy profile compared with the tumour bulk.

In conclusion, using an integrated analysis combining ploidy with copy number aberrations and phenotypic marker expression, we provide detailed evidence of inter- and intra-tumoural clonal heterogeneity in GBMs. These data further our knowledge on heterogeneity and clonal evolution in GBM, which are crucial parameters that may impact GBM progression and treatment responses.

## Electronic supplementary material

Below is the link to the electronic supplementary material.

Supplementary material 1 (PDF 1236 kb)

Supplementary material 2 (DOCX 38 kb)
