# Supplementary figures and images for: Glioblastomas are composed of genetically divergent clones with distinct tumourigenic potential and variable stem cell-associated phenotypes
Source: Acta Neuropathol. 2013 Oct 24;127(2):203–19. doi: 10.1007/s00401-013-1196-4 (PMC3895194; doi:10.1007/s00401-013-1196-4)

# Suppl. Fig. 1

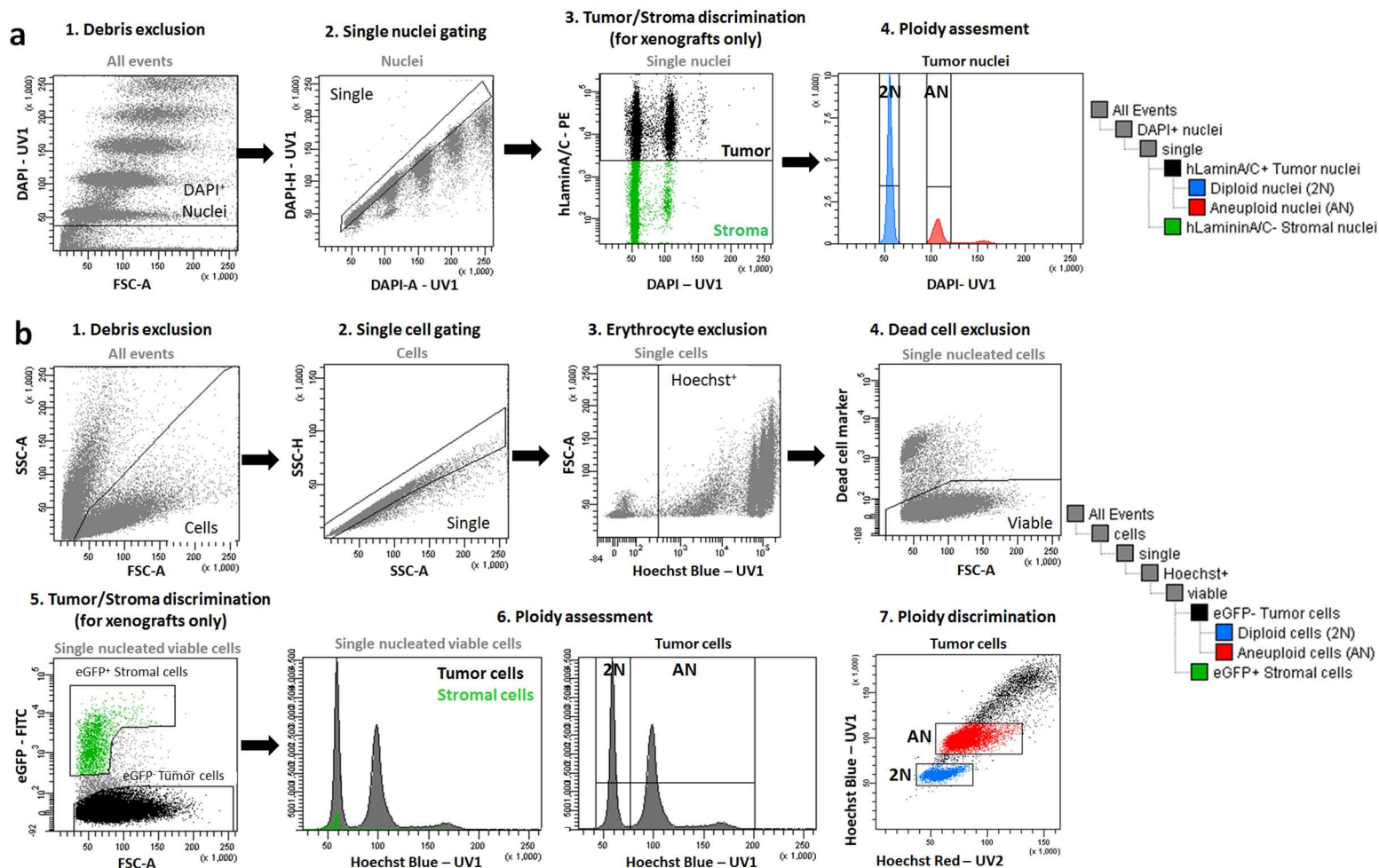

Suppl. Fig 2

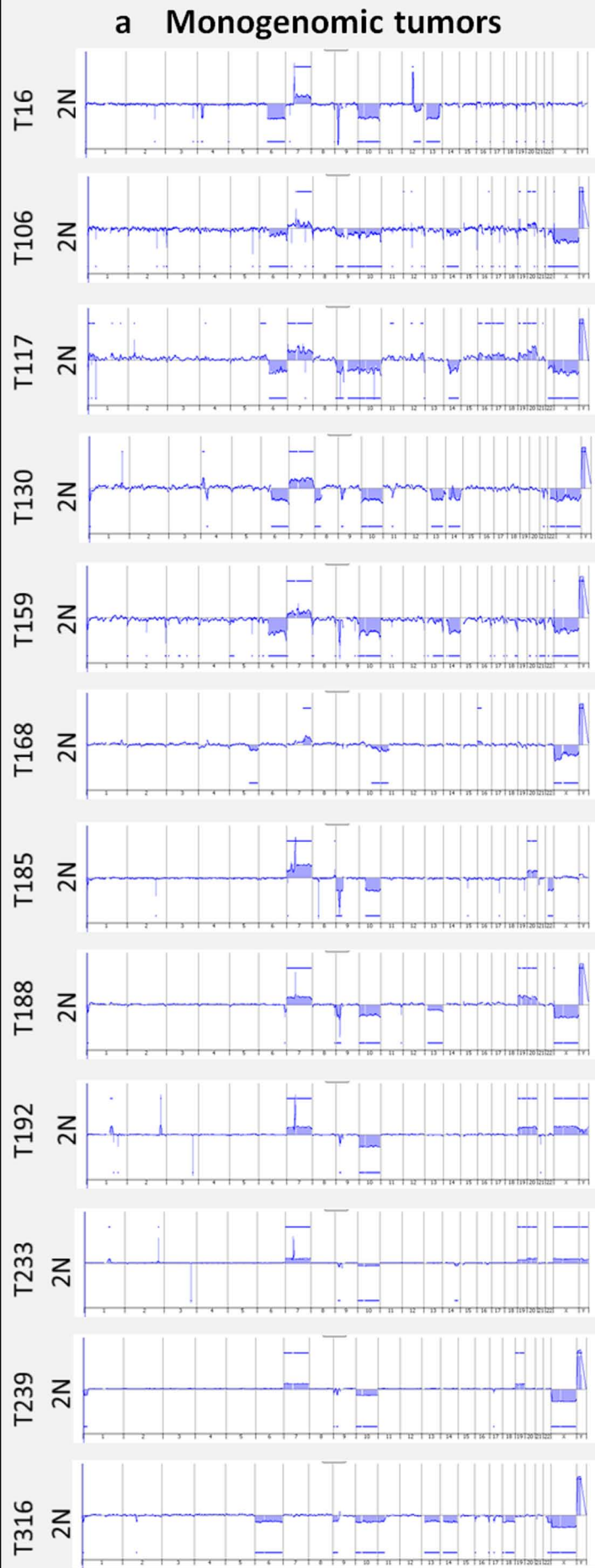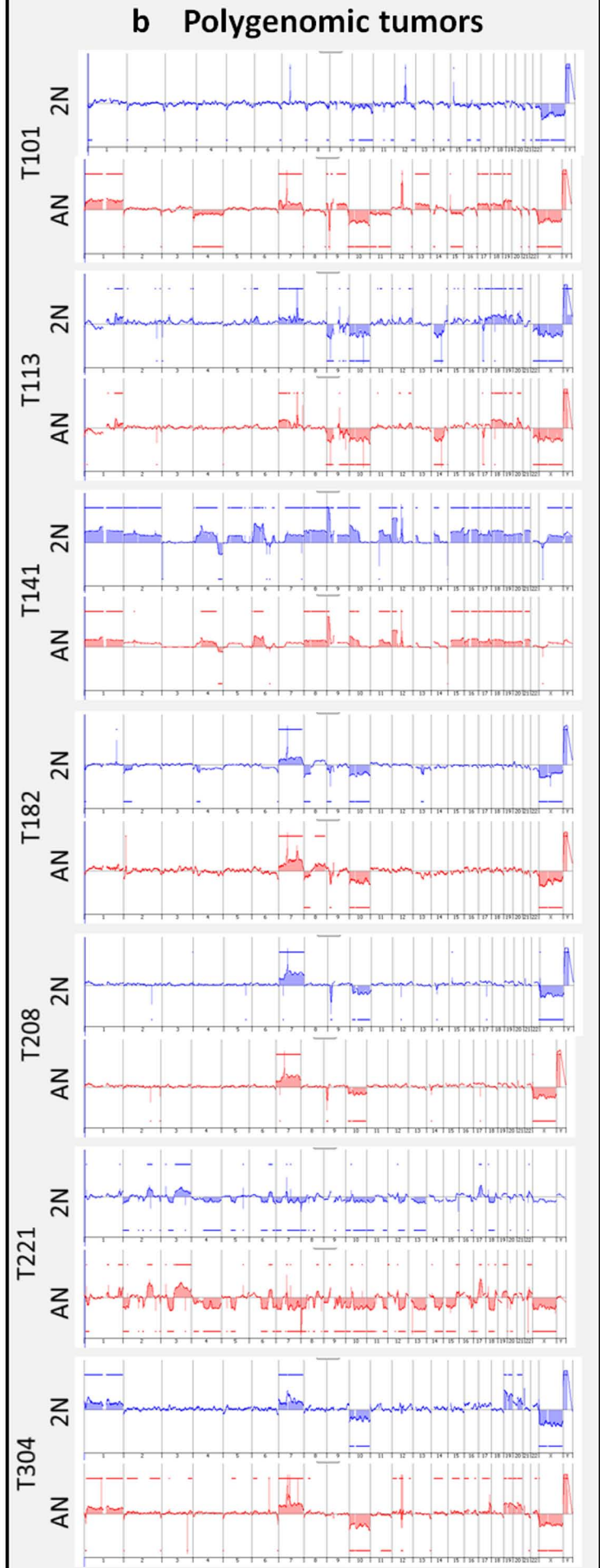

# Suppl. Fig 3

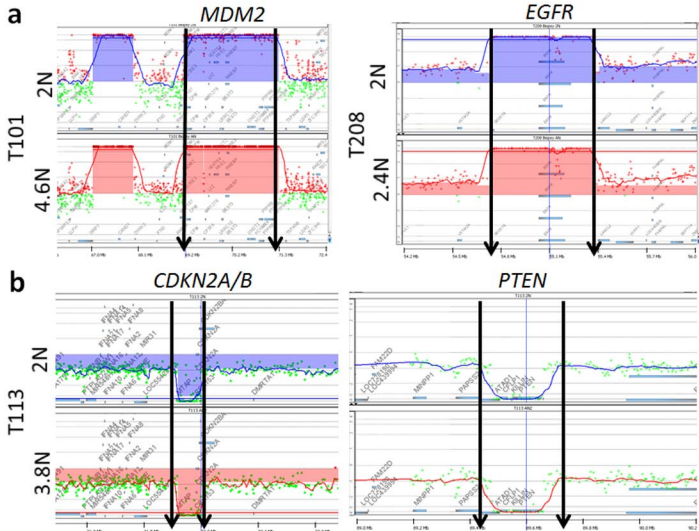

# Suppl. Fig 4

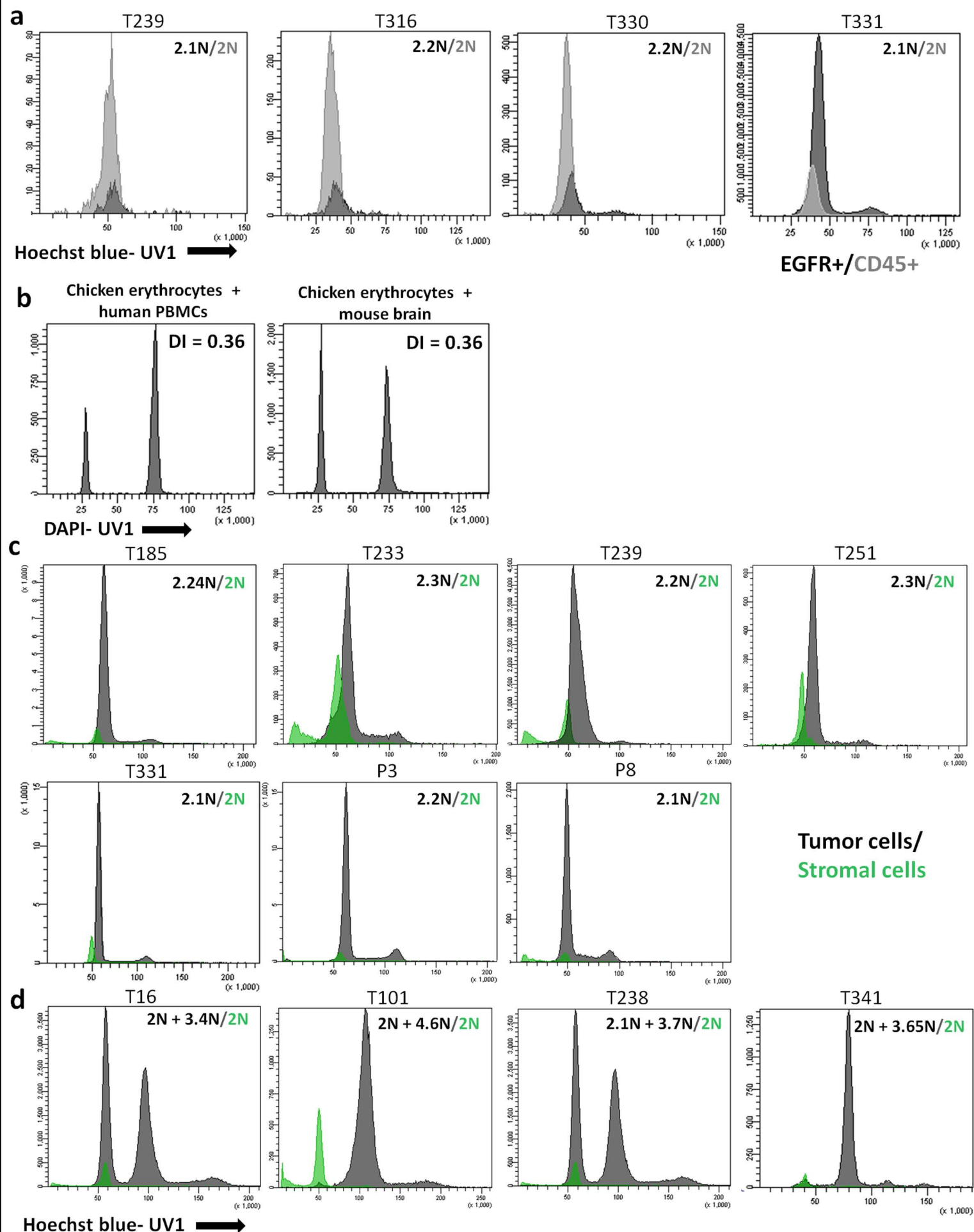

Supplement: Supplementary file 1 — Supplementary material 1 (PDF 1236 kb) [file 401_2013_1196_MOESM1_ESM.pdf]
