# Supplementary material for: Glioblastomas are composed of genetically divergent clones with distinct tumourigenic potential and variable stem cell-associated phenotypes
Source: Acta Neuropathol. 2013 Oct 24;127(2):203–19. doi: 10.1007/s00401-013-1196-4 (PMC3895194; doi:10.1007/s00401-013-1196-4)
Supplement: Supplementary file 2 — Supplementary material 2 (DOCX 38 kb) [file 401_2013_1196_MOESM2_ESM.docx]

**ELECTRONIC SUPPLEMENTARY MATERIAL**

**Glioblastomas are composed of genetically divergent clones with distinct tumorigenic potential and variable stem cell-associated phenotypes**

**AUTHORS:**

Daniel Stieber^1*^, Anna Golebiewska^1*^, Lisa Evers^2^, Elizabeth Lenkiewicz^2^, Nicolaas H.C. Brons^3^, Nathalie Nicot^4^, Anaïs Oudin^1^, Sébastien Bougnaud^1^, Frank Hertel^5^, Rolf Bjerkvig^1,6^, Laurent Vallar^4^, Michael T. Barrett^2^ and Simone P. Niclou^1^.

* These authors contributed equally to the work

**AFFILIATIONS:**

^1^NorLux Neuro-Oncology Laboratory, Department of Oncology, ^3^Core Facility Flow Cytometry, ^4^Genomics Research Unit, Centre de Recherche Public de la Santé (CRP-Santé), Luxembourg, Luxembourg; ^2^Clinical Translational Research Division, Translational Genomics Research Institute, Scottsdale, AZ, USA ; ^5^Department of Neurosurgery, Centre Hospitalier de Luxembourg, Luxembourg; ^6^NorLux Neuro-Oncology, Department of Biomedicine, University of Bergen, Norway.

**CONTACT INFORMATION:**

Simone P. Niclou, PhD

Centre de Recherche Public de la Santé (CRP-Santé)

Department of Oncology

NorLux Neuro-Oncology Laboratory

84, Val Fleuri, L- 1526 Luxembourg

tel. + 352-26970-273

fax.+ 352-26970-390

[simone.niclou@crp-sante.lu](mailto:simone.niclou@crp-sante.lu)

Electronic supplementary material includes 4 Figures and 3 Tables. **Suppl. Fig. 1**: Gating strategy for ploidy analysis. **Suppl. Fig. 2**: array CGH profile of purified tumor fractions. **Suppl. Fig. 3**: Analysis of chromosomal breakpoints in polygenomic tumors. **Suppl. Fig. 4**: Hoechst-based ploidy analysis in patient biopsies and orthotopic xenografts. **Suppl. Table 1**: List of GBM patient biopsies analysed at the ploidy level; **Suppl. Table 2**: Ploidy analysis of patient biopsies and their respective xenografts; **Suppl. Table 3**: List of antibodies.

**SUPPLEMENTAL FIGURE LEGENDS**

**Suppl. Fig. 1** Gating strategy for ploidy analysis (Related to **Fig. 1-5**). The step by step gating strategy for ploidy analysis is shown for the intracranial T16 xenograft in NOD/SCID mice. The same strategy was used for all subsequent experiments. **a Gating strategy for ploidy analysis in isolated nuclei.** (**1)** Intact nuclei were distinguished from debris and erythrocytes on the flow cytometric profile based on positive DAPI staining. (**2)** Nuclei doublets and aggregates were gated out based on their properties displayed on the DAPI area (DAPI-A) versus height (DAPI-H) dot plot. (**3)** In xenografts, human tumor cells were recognized as the human-specific lamin A/C positive population (‘black’) (**4)** Tumor nuclei displayed different levels of ploidy (blue = 2N and red = AN nuclei)**.** For the aneuploid clones overlapping with the diploid peaks in the G1 (2N) or G_2_/M (4N) cell cycle phase only the non-overlapping fraction of the peaks was gated for sorting. **b Gating strategy for ploidy analysis in viable cells.** (**1)** Cells were distinguished from debris on the flow cytometric profile based on the Forward Scatter (FSC) and Side Scatter (SSC). (**2)** Cell doublets and aggregates were gated out based on their properties displayed on the SSC area (SSC-A) versus height (SSC-H) dot plot. (**3)** For cell suspensions from primary tissues and tumors, erythrocytes were excluded based on the negative signal in the Hoechst blue channels. (**4)** Dead cells were discriminated by a dead cell marker. (**5)** In xenografts, human tumor cells were recognized as the eGFP^-^ population compared to the eGFP^+^ mouse stromal host cells. (**6)** Tumor (black) and host (green) cell population display on the Hoechst dot plot shows that tumor cells can display different levels of ploidy: diploid (2N, blue) and aneuploid (AN, red) cells. Mouse stromal cells were used as a diploid control. As a subpopulation of eGFP^+^ stromal cells is able to efflux the Hoechst dye [[1](#_ENREF_1)] the first clear distinct Hoechst peak was used as the diploid control. (**7**) Tumor cells with different ploidies were discriminated for sorting based on the Hoechst signal detected in Hoechst red versus Hoechst blue channels

**Suppl. Fig. 2** aCGH profile of purified tumor fractions (Related to **Fig. 1b**). Genome View aCGH profiles were obtained for sorted nuclei of each DAPI peak detected in patient biopsies. **a** aCGH profiles of monogenomic patient biopsies. **b** aCGH profiles of sorted diploid (blue) and aneuploidy (red) fractions of polygenomic patient biopsies. Each fraction displayed glioma specific tumor rearrangements, confirming the presence of tumor cells in each sorted population

**Suppl. Fig. 3** Analysis of chromosomal breakpoints in distinct clones of polygenomic tumors (Related to **Fig. 2e**). Analysis of chromosomal breakpoints in polygenomic tumors. Chromosomal breakpoints present in divergent clones within the same patient biopsies were identical. **a** Examples of focal amplicons (MDM2 in T101; EGFR in T208). **b** Examples of homozygous deletions (CDKN2A/B and PTEN in T113)

**Suppl. Fig. 4** Hoechst-based ploidy analysis in patient biopsies and orthotropic xenografts (Related to **Fig. 2a** and **Fig. 3**). **a** Related to **Fig. 2a**, Hoechst-based profiles of EGFR^+^ tumor cells (‘black’) and CD45^+^ hematopoietic stromal cells (‘grey’) are shown for GBM patient biopsies. Ploidy analysis of EGFR^+^ tumor cells showed a pseudodiploid peak with a significant shift in the DNA content compared to the CD45^+^ diploid control. **b** Related to **Fig. 3,** DNA content analysis in human peripheral blood mononuclear cells (PBMCs) and mouse brain is comparable. The DNA-index (DI) compared to chicken erythrocytes was 0.36 in both cases. **c** Ploidy analysis of monogenomic tumors in respective xenografts. **d** Ploidy analysis of polygenomic tumors in respective xenografts. For **c-d** eGFP^+^ NOD/SCID mice were used. eGFP^+^ mouse cells (green) were used as a diploid control for ploidy calculations of eGFP^-^ tumor cells (black)

**SUPPLEMENTARY TABLES**

**Suppl. Table 1** List of GBM patient biopsies analysed for ploidy level. Ploidy levels were detected by DAPI-based staining in nuclei isolated from primary GBM patient biopsies. Assuming that stromal cells were present in each patient biopsy the first peak recognised was considered as 2N control

| **GBM patient biopsy** | **Diploid** | **Aneuploid 1** | **Aneuploid 2** | **Recurrence** | **Sex** | **Age** |
| --- | --- | --- | --- | --- | --- | --- |
| T16 | 2N |  |  |  | F | 76 |
| T101 | 2N | 4,6N |  |  | M | 60 |
| T106 | 2N |  |  |  | M | 57 |
| T113 | 2N | 3,2N | 3,8N |  | M | 56 |
| T117 | 2N |  |  |  | M | 57 |
| T130 | 2N |  |  |  | M | 62 |
| T141 | 2N | 3,3N |  |  | F | 35 |
| T145 | 2N | 4,8N | 9,3N |  | M | 64 |
| T159 | 2N |  |  |  | M | 69 |
| T168 | 2N |  |  |  | M | 58 |
| T176 | 2N | 3,6N |  |  | M | 50 |
| T182 | 2N | 3.9N |  |  | M | 46 |
| T185 | 2N |  |  |  | F | 76 |
| T188 | 2N |  |  |  | M | 68 |
| T192 | 2N |  |  |  | F | 42 |
| T200 | 2N |  |  |  | M | 52 |
| T207 | 2N |  |  |  | F | 66 |
| T208 | 2N | 2,4N |  |  | M | 66 |
| T221 | 2N | 2,6N |  |  | M | 71 |
| T226 | 2N |  |  | *T188 | M | 69 |
| T233 | 2N |  |  | *T192 | F | 42 |
| T238 | 2N | 3,5N |  |  | M | 42 |
| T239 | 2N |  |  |  | M | 80 |
| T251 | 2N |  |  | *T192/T233 | F | 43 |
| T265 | 2N | 3,6N |  |  | F | 80 |
| T291 | 2N |  |  |  | M | 77 |
| T293 | 2N |  |  |  | M | 42 |
| T304 | 2N | 3,9N |  |  | M | 53 |
| T316 | 2N |  |  |  | M | 51 |
| T328 | 2N |  |  |  | F | 71 |
| T330 | 2N |  |  |  | M | 70 |
| T331 | 2N |  |  |  | M | 81 |
| T341 | 2N | 3,2N |  |  | F | 75 |
| T344 | 2N |  |  | *T316 | M | 51 |
| T346 | 2N | 4N |  | *T304 | M | 53 |
| T400 | 2N |  |  | *T200 | M | 56 |

* Biopsies from recurrent GBM tumors

**Suppl. Table 2** List of xenotransplanted patient biopsies. A number of patient biopsies were used for subsequent spheroid-based xenotransplantations in NOD/Scid mice. Ploidy was analyzed in tumor cells of the xenografts and compared to the ploidy profile in equivalent patient biopsies. With exception for T16 patient biopsies all clones were detected at the same level in xenografts compared to biopsies. As T16 patient biopsy used for the analysis was relatively small we assumed that the aneuploid clone was present only in the tissue used for spheroid derivation.

| Patient biopsy | Patient biopsy ploidy | Xenograft tumor cell /Cell line ploidy | Mean xenograft development (days) |
| --- | --- | --- | --- |
| **Spheroid-based xenografts** | | | |
| T16 | 2N | 2N + 3.4N | 140 +/- 15 (G1) – 74 +/- 2 (G5); n=16 |
| T101 | 2N + 4.6N | 2N + 4.6N | 111 +/- 2.4 (G1) – 91 +/ 0.4 (G6); n=10 |
| T185 | 2N | 2N | 142 +/- 0.5; n=6 |
| T233 | 2N | 2N | 129 +/- 10; n=6 |
| T238 | 2N + 3.7N | 2N + 3.7N | 139 +/- 1 (G1) ; n=3 – 84.4 +/5 (G3); n=5 |
| T239 | 2N | 2N | 140 +/- 6; n=6 |
| T251 | 2N | 2N | 103 +/- 7.5; n=7 |
| T331 | 2N | 2N | 142 +/- 8 ; n=3 |
| T341 | 2N + 3.65N | 2N + 3.65N | 59.5 +/- 0.3 ; n=4 |
| P3 | na | 2N | 37 +/- 4; n = 8 |
| P8 | na | 2N | 64.5 +/- 0.5; n = 8 |
| **Glioma stem-like cell lines** | | | |
| NCH421k | na | 3.46N | 71 +/- 0.8; n = 21 |
| NCH465 | na | 3N | na |
| NCH601 | na | 3.7N | na |
| NCH660 | na | 3.3N | na |
| NCH644 | na | 3.5N | 31.5 +/- 1; n = 14 |
| TB101 | na | 3.1N | na |
| TB107 | na | 2N + 3.6N | na |
| **Classical glioma adherent cell lines** | | | |
| U87 | na | 3.7N | 24.4 +/- 0.8 ; n=9 |
| U251 | na | 2.26N | 41.8 +/- 0.2 ; n=5 |
| U373 | na | 2.84N | na |

na= non-assessed

G= xenograft generation during serial transplantation

**Suppl. Table 3** List of antibodies used in the study

| **Epitope** | **Conjugate** | **Clone** | **Supplier** | **Concentration used/test*** |
| --- | --- | --- | --- | --- |
| A2B5 | APC/PE | 105-HB29 | Miltenyi | 10µl/test |
| CD15/SSEA-1 | Alexa Fluor 647 | MC-480 | Biolegend | 5µl/test |
| CD15/SSEA-1 | PE | MEM-158 | Immunotools | 10µl/test |
| CD44 | PE-Cy7 | IM7 | eBioscience | 1.2µl/test |
| CD45 | FITC | 15D9 | Immunotools | 10µl/test |
| CD56 | PE-Cy7 | N-CAM | BD Bioscience | 5µl/test |
| CD133 | PE /APC | 293C3/AC133 | Miltenyi | 10µl/test |
| EGFR | PE | EGFR.1 | BD Bioscience | 20µl/test |
| NG2 | PE | LHM-2 | R&D | 10µl/test |
| laminA/C | PE | sc-7292 | Santa Cruz | 20µl/test |

*Flow cytometry test: 10^6^ cells/100µl

**SUPPLEMENTARY REFERENCES**

1. Golebiewska A, Bougnaud S, Stieber D, Brons NH, Vallar L, Hertel F, Klink B, Schrock E, Bjerkvig R, Niclou SP (2013) Side population in human glioblastoma is non-tumorigenic and characterizes brain endothelial cells. Brain : a journal of neurology 136 (Pt 5):1462-1475. doi:10.1093/brain/awt025
